# A randomized phase 2 study on demeclocycline in patients with mild-to-moderate COVID-19

**DOI:** 10.1038/s41598-023-41051-2

**Published:** 2023-08-23

**Authors:** Kota Iwahori, Takuro Nii, Norihiko Yamaguchi, Takahiro Kawasaki, Satomi Okamura, Kazuki Hashimoto, Takanori Matsuki, Kazuyuki Tsujino, Keisuke Miki, Akio Osa, Sho Goya, Kinya Abe, Masahide Mori, Yoshito Takeda, Tomomi Yamada, Hiroshi Kida, Atsushi Kumanogoh

**Affiliations:** 1 Department of Respiratory Medicine and Clinical Immunology, Graduate School of Medicine, Osaka University, Suita, Osaka Japan; 2 Department of Clinical Research in Tumor Immunology, Graduate School of Medicine, Osaka University, Suita, Osaka Japan; 3 Department of Respiratory Medicine, National Hospital Organization Osaka Toneyama Medical Center, Toyonaka, Osaka Japan; 4 Department of Respiratory Medicine, Kinki Central Hospital of Mutual Aid Association of Public School Teachers, Itami, Hyogo Japan; 5 Department of Medical Innovation, Osaka University Hospital, Suita, Osaka Japan; 6 Department of Internal Medicine, Toyonaka Municipal Hospital, Toyonaka, Osaka Japan; 7 Department of Thoracic Oncology, National Hospital Organization Osaka Toneyama Medical Center, Toyonaka, Osaka Japan; 8 Department of Immunopathology, World Premier International Research Center Initiative (WPI), Immunology Frontier Research Center (IFReC), Osaka University, Suita, Osaka Japan; 9 Integrated Frontier Research for Medical Science Division, Institute for Open and Transdisciplinary Research Initiatives (OTRI), Osaka University, Suita, Osaka Japan; 10 Center for Infectious Diseases for Education and Research (CiDER), Osaka University, Suita, Osaka Japan; 11 Japan Agency for Medical Research and Development–Core Research for Evolutional Science and Technology (AMED–CREST), Osaka University, Suita, Osaka Japan; 12 Center for Advanced Modalities and DDS (CAMaD), Osaka University, Suita, Osaka Japan

**Keywords:** SARS-CoV-2, Viral infection

## Abstract

Tetracyclines exhibit anti-viral, anti-inflammatory, and immunomodulatory activities via various mechanisms. The present study investigated the efficacy and safety of demeclocycline in patients hospitalized with mild-to-moderate COVID-19 via an open-label, multicenter, parallel-group, randomized controlled phase 2 trial. Primary and secondary outcomes included changes from baseline (day 1, before the study treatment) in lymphocytes, cytokines, and SARS-CoV-2 RNA on day 8. Seven, seven, and six patients in the control, demeclocycline 150 mg daily, and demeclocycline 300 mg daily groups, respectively, were included in the modified intention-to-treat population that was followed until day 29. A significant change of 191.3/μL in the number of CD4^+^ T cells from day 1 to day 8 was observed in the demeclocycline 150 mg group (95% CI 5.1/μL–377.6/μL) (p = 0.023), whereas that in the control group was 47.8/μL (95% CI − 151.2/μL to 246.8/μL), which was not significant (p = 0.271). The change rates of CD4^+^ T cells negatively correlated with those of IL-6 in the demeclocycline-treated groups (R = − 0.807, p = 0.009). All treatment-emergent adverse events were of mild-to-moderate severity. The present results indicate that the treatment of mild-to-moderate COVID-19 patients with demeclocycline elicits immune responses conducive to recovery from COVID-19 with good tolerability.

**Trial registration**: This study was registered with the Japan Registry of Clinical Trials (Trial registration number: jRCTs051200049; Date of the first registration: 26/08/2020).

## Introduction

Since the outbreak of the coronavirus disease 2019 (COVID-19) pandemic, the development of vaccines and therapeutic agents has mitigated its severity and mortality. mRNA vaccines, such as BNT162b2 and mRNA-1273^1,2^, adenoviral vector vaccines, including ChAdOx1 nCoV-19 and Ad26.COV2.S^3,4^, and the recombinant nanoparticle vaccine NVX-CoV2373^5^ have been developed as COVID-19 vaccines to confer immunity against severe acute respiratory syndrome coronavirus 2 (SARS-CoV-2) infection. Antiviral agents, such as remdesivir, molnupiravir, and the combination of nirmatrelvir and ritonavir^6–8^, as well as neutralizing antibodies, including sotrovimab, the combination of casirivimab and imdevimab, and the combination of bamlanivimab and etesevimab^9–12^, have been shown to reduce the severity and mortality of COVID-19.

In addition to vaccines, antiviral agents, and neutralizing antibodies, which are specific to the SARS-CoV-2 virus, repurposed immunomodulatory agents, such as dexamethasone, balicitinib, tocilizumab, and sarilumab, have been shown to mitigate the severity and mortality of COVID-19^13–15^. These immunomodulatory agents, which include a glucocorticoid (dexamethasone), Janus kinase inhibitor (balicitinib), and interleukin-6 receptor antagonists (tocilizumab and sarilumab), exert immunosuppressive effects and reduce inflammatory responses in patients with moderate and severe COVID-19. Conversely, in the early and mild phases of COVID-19, antiviral T cell responses play a crucial role in recovery^16^. During SARS-CoV-2 infection, CD4^+^ and CD8^+^ T cells coordinate humoral and cellular immune responses. Early in the COVID-19 pandemic, initial studies indicated that the peripheral CD8^+^ T cells of recovered COVID-19 patients significantly increased within seven days of early infection^17,18^. Although antiviral T cell responses are critical to recovery from COVID-19, there are currently no therapeutic agents that augment the antiviral T cell responses of COVID-19 patients.

Tetracyclines exhibit anti-viral and anti-inflammatory activities through multiple mechanisms^19^. In vitro studies demonstrated that doxycycline exhibited antiviral activity against SARS-CoV-2^20^ and anti-inflammatory activity by inhibiting nitric oxide production^21^ and matrix metalloproteinase-9^22^. However, clinical trials on doxycycline as a treatment for suspected COVID-19 did not show reductions in the time to recovery, hospital admissions, or deaths related to COVID-19^23^. Moreover, we found that, in addition to their previously reported anti-inflammatory effects, tetracyclines enhanced antigen-specific T cell responses at lower concentrations than those typically used as antibiotics^24^. These immunomodulatory properties of tetracyclines prompted us to conduct a clinical study on the immune responses of COVID-19 patients treated with tetracyclines. In the present study, we selected demeclocycline, a tetracycline that is less frequently used as an antibiotic. We focused on immunological responses, including changes in lymphocytes and cytokines, before and after treatment with low-dose demeclocycline.

## Results

### Disposition of patients

**Figure 1:**
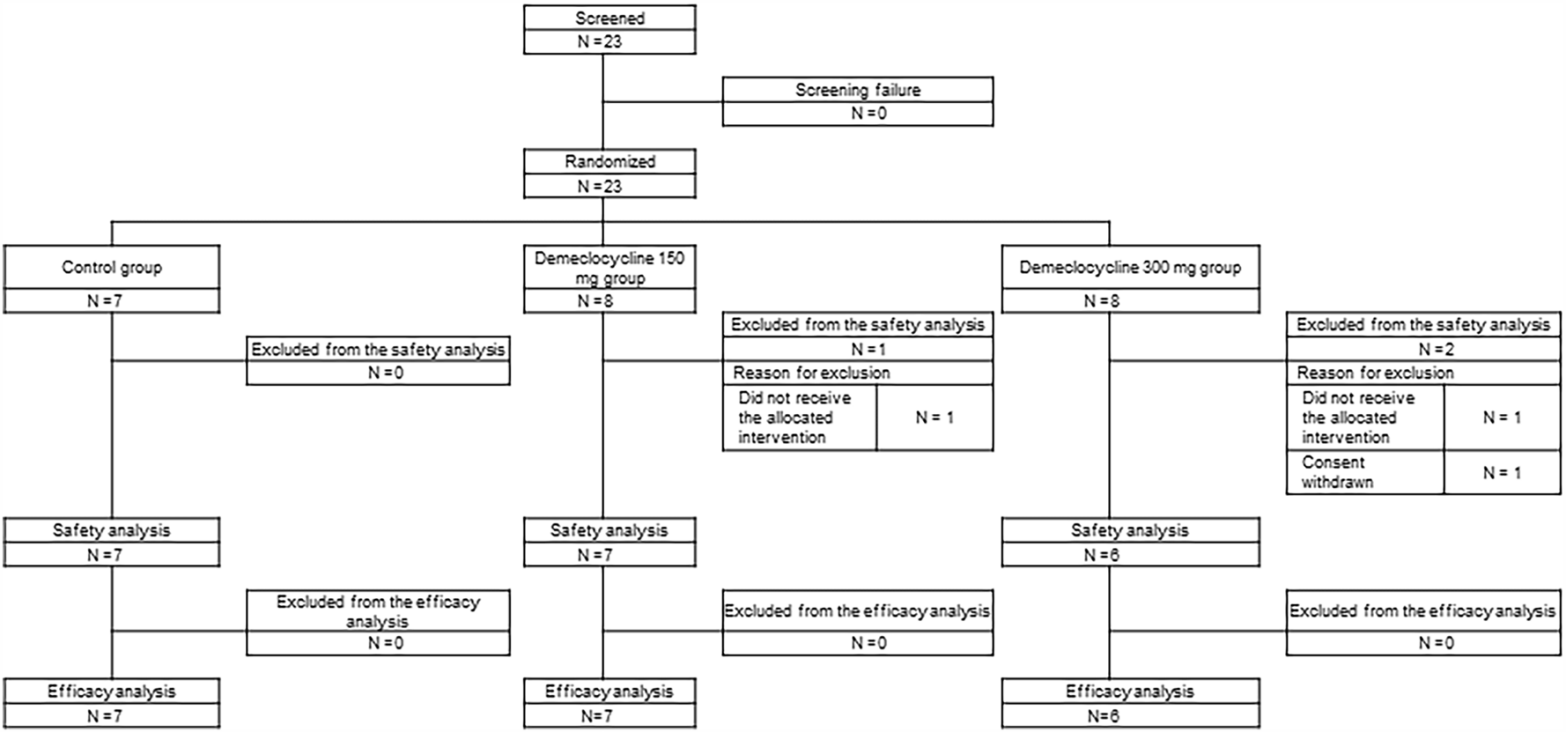
Flow diagram.

This open-label, multicenter, parallel-group, randomized controlled trial was conducted between September 1, 2020 and March 31, 2022 at four institutes in Osaka, Japan. The last entry was on September 22, 2021, when the delta variant of SARS-CoV-2 was prevalent in Japan. Twenty-three hospitalized patients who provided informed consent and were screened were randomized into three groups: a control group (n = 7), demeclocycline 150 mg daily group (n = 8), and demeclocycline 300 mg daily group (n = 8) (Fig. 1). Among the 23 randomized patients, one in the demeclocycline 150 mg group and one in the demeclocycline 300 mg group did not receive the study drug and, thus, were excluded from efficacy and safety analyses. Furthermore, one patient in the demeclocycline 300 mg group was excluded from the safety analysis due to the withdrawal of consent. Therefore, 20 patients (control group (n = 7), demeclocycline 150 mg group (n = 7), and demeclocycline 300 mg group (n = 6)) were included in the modified intention-to-treat (mITT) population.

### Baseline characteristics of patients

The baseline characteristics of the 20 patients in the mITT population were similar across groups (Table 1). The lowest oxygen saturation (SpO_2_) concentration was 95% at room air in each group. All patients in each group were diagnosed with pneumonia by chest imaging (chest X-ray or computed tomography). The most frequent coexisting conditions were hypertension (20.0%) and diabetes mellitus (20.0%). The median duration from symptom onset to the initiation of the study treatment was 7.5 days (range, 4 to 19 days).

**Table 1:** Baseline clinical characteristics (mITT population).

	Total	Control group	Demeclocycline 150 mg group	Demeclocycline 300 mg group
Number of patients	20	7	7	6
Age				
Mean	57.6	65.0	53.1	54.0
SD	9.6	8.9	9.1	6.0
Range	42–72	46–72	42–69	48–62
Sex				
Male sex, N (%)	12 (60.0%)	3 (42.9%)	4 (57.1%)	5 (83.3%)
Coexisting conditions, N (%)				
Bronchial ectasia	1 (5.0%)	1 (14.3%)	0 (0.0%)	0 (0.0%)
Bronchial asthma	3 (15.0%)	0 (0.0%)	3 (42.9%)	0 (0.0%)
Hypertension	4 (20.0%)	2 (28.6%)	2 (28.6%)	0 (0.0%)
Osteoporosis	1 (5.0%)	0 (0.0%)	1 (14.3%)	0 (0.0%)
Dyslipidemia	1 (5.0%)	1 (14.3%)	0 (0.0%)	0 (0.0%)
Mediastinal tumor	1 (5.0%)	0 (0.0%)	0 (0.0%)	1 (16.7%)
Latent tuberculosis infection	1 (5.0%)	0 (0.0%)	0 (0.0%)	1 (16.7%)
Mitral insufficiency	1 (5.0%)	0 (0.0%)	1 (14.3%)	0 (0.0%)
History of cerebral infarction	1 (5.0%)	0 (0.0%)	1 (14.3%)	0 (0.0%)
History of tuberculosis	1 (5.0%)	1 (14.3%)	0 (0.0%)	0 (0.0%)
Gout	1 (5.0%)	0 (0.0%)	0 (0.0%)	1 (16.7%)
Diabetes mellitus	4 (20.0%)	2 (28.6%)	2 (28.6%)	0 (0.0%)
Sinusitis	2 (10.0%)	1 (14.3%)	0 (0.0%)	1 (16.7%)
Chronic urticaria	1 (5.0%)	0 (0.0%)	1 (14.3%)	0 (0.0%)
Pneumonia				
No	0 (0.0%)	0 (0.0%)	0 (0.0%)	0 (0.0%)
Yes	20 (100.0%)	7 (100.0%)	7 (100.0%)	6 (100.0%)
SpO_2_				
Mean	96.5	96.7	95.9	96.8
SD	1.2	1.3	0.9	1.3
Range	95–99	95–98	95–97	95–99
SARS-CoV-2 PCR or antigen test				
Negative	0 (0.0%)	0 (0.0%)	0 (0.0%)	0 (0.0%)
Positive	20 (100.0%)	7 (100.0%)	7 (100.0%)	6 (100.0%)
SARS-CoV-2 PCR	18	6	7	5
SARS-CoV-2 antigen	2	1	0	1
Time from onset to the initiation of the study treatment (days)				
Median	7.5	7.0	9.0	5.0
Range	4–19	4–14	5–19	4–14

mITT: modified intention-to-treat.

### Preventing the exacerbation of COVID-19

**Figure 2:**
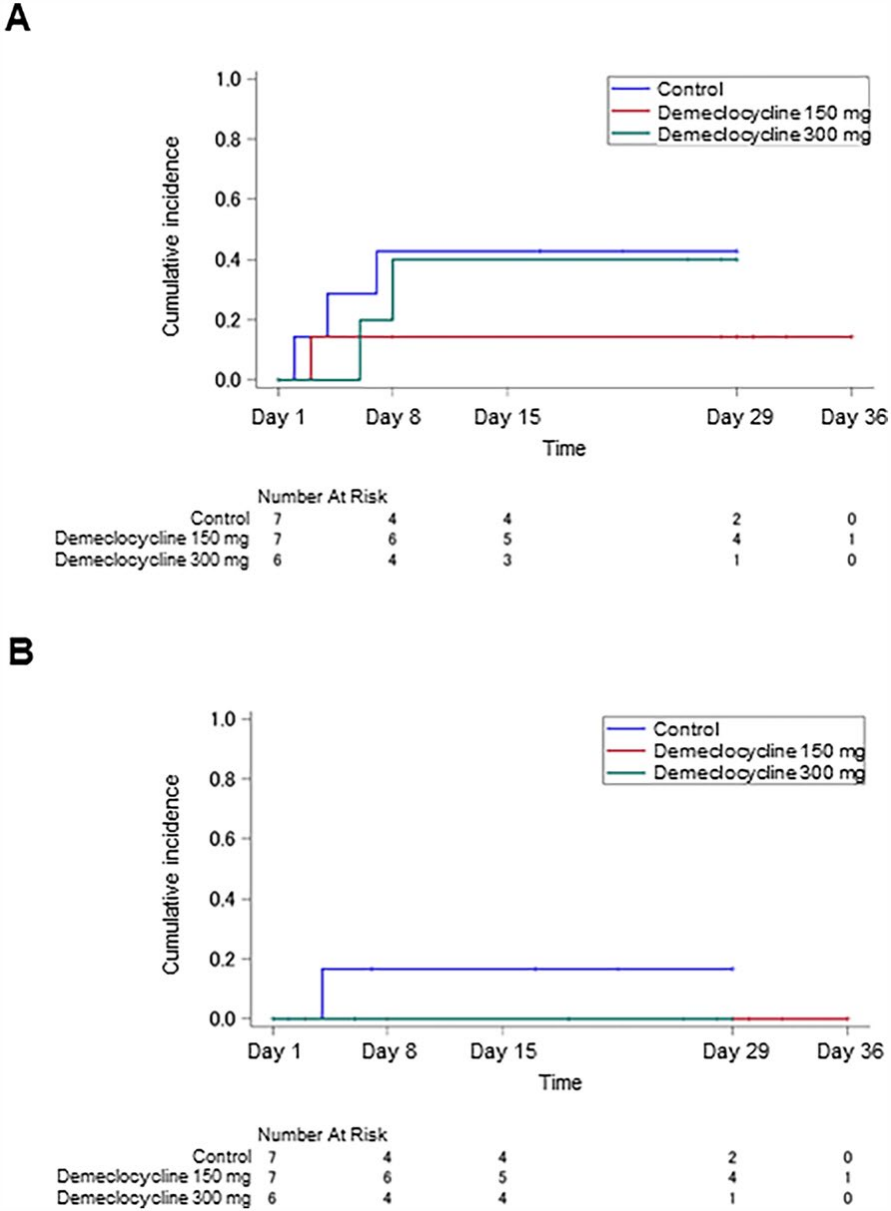
(**A**) Kaplan–Meier curves for the cumulative incidence of the dexamethasone treatment due to COVID-19 exacerbations. (**B**) Kaplan–Meier curves for the cumulative incidence of continuous oxygen therapy due to respiratory failure in COVID-19.

Kaplan–Meier estimates of the cumulative incidence of dexamethasone treatment due to COVID-19 exacerbations by day 8 were 42.9% in the control group (95% CI 7.6% to 75.7%), 14.3% in the demeclocycline 150 mg group (95% CI 0.5% to 49.1%), and 40.0% in the demeclocycline 300 mg group (95% CI 3.1% to 78.6%) (Fig. 2A). Subsequently, on or after day 8, there were no changes in the cumulative incidence of dexamethasone treatment within each group. Furthermore, no significant differences were observed in the time to dexamethasone treatment between the control group and demeclocycline 150 and 300 mg groups (p = 0.139 and 0.388, respectively). To minimize the effects of T cell responses, the concurrent administration of dexamethasone was prohibited during the course of the study. Consequently, the study treatment was discontinued upon the administration of dexamethasone. Concurrent treatments in each group are shown in Supplementary Table 1.

Kaplan–Meier estimates of the cumulative incidence of continuous oxygen therapy due to respiratory failure in COVID-19 by day 8 were 16.7% in the control group (95% CI 0.5% to 54.9%) and 0.0% in the demeclocycline treatment groups (Fig. 2B). Thereafter, on or after day 8, there were no changes in the cumulative incidence of continuous oxygen therapy within each group. Moreover, no significant differences were observed in the time to continuous oxygen therapy between the control group and demeclocycline 150 and 300 mg groups (p = 0.159 and 0.181, respectively).

Average changes in oxygen saturation measured by pulse oximetry (SpO_2_) were 0.7% in the demeclocycline 150 mg group, 0.2% in the demeclocycline 300 mg group, and 0.2% in the control group (Supplementary Fig. 1). Differences in the average change in SpO_2_ from the control group to the demeclocycline 150 and 300 mg groups were 0.5% (95% CI − 0.8% to 1.8%) and 0.0% (95% CI − 2.2% to 2.3%), respectively, which were not significant (p = 0.205 and p = 0.487, respectively). The National Early Warning Score (NEWS) and seven-category ordinal scale are shown in Supplementary Tables 2, 3, and Supplementary Fig. 2. The highest rate (85.7%) of patients discharged without treatment by day 29 was observed in the demeclocycline 150 mg group.

**Table 2:** Negative conversion of saliva SARS-CoV-2 RNA levels on day 8.

	Control group	Demeclocycline 150 mg group	Demeclocycline 300 mg group
Number of patients	6	7	4
Negative conversion on day 8, N (%)	1 (16.7%)	3 (42.9%)	2 (50.0%)
Without negative conversion, N (%)	5 (83.3%)	4 (57.1%)	2 (50.0%)
Detection of SARS-CoV-2 RNA on day 8	3	3	0
The study treatment was discontinued before day 8 due to dexamethasone or oxygen therapy	2	1	2

**Table 3:** Changes in and change rates of T cells from day 1 to day 8 of the study treatment.

Arm		CD8^+^ T cells				CD4^+^ T cells			
		Day 1 (/µL)	Day 8 (/µL)	Change (/µL)	Change rate (%)	Day 1 (/µL)	Day 8 (/µL)	Change (/µL)	Change rate (%)
Control		430	538	108	25.1	502	658	156	31
		646	674	28	4.3	420	282	− 138	− 33
		223	247	24	10.8	324	261	− 63	− 19
		305	322	17	5.6	754	781	27	4
		183	503	320	174.9	143	400	257	180
		242	–			740	–		
		409	–			583	–		
	Average			99.4 (N = 5)	44.1 (N = 5)			47.8 (N = 5)	32.4 (N = 5)
Demeclocycline 150 mg		405	477	72	17.8	361	354	− 7	− 2
		405	352	− 53	− 13.1	681	705	24	4
		217	322	105	48.4	453	597	144	32
		384	464	80	20.8	182	384	202	111
		292	519	227	77.7	393	844	451	115
		245	471	226	92.2	404	738	334	83
		318	–			652	–		
	Average			109.5 (N = 6)	40.7 (N = 6)			191.3 (N = 6)	57.0 (N = 6)
Demeclocycline 300 mg		505	463	− 42	− 8.3	439	397	− 42	− 10
		321	460	139	43.3	522	751	229	44
		226	536	310	137.2	339	919	580	171
		524	–			597	–		
		323	–			333	–		
		234	–			326	–		
	Average			135.7 (N = 3)	57.4 (N = 3)			255.7 (N = 3)	68.5 (N = 3)

### SARS-CoV-2 viral RNA level

Saliva SARS-CoV-2 RNA turned negative on day 8 in 42.9% (3/7) of patients in the demeclocycline 150 mg group and 50.0% (2/4) in the demeclocycline 300 mg group, in contrast to only 16.7% (1/6) in the control group (as shown in Table 2 and Supplementary Fig. 3). There were no significant differences in the rate of the negative conversion of SARS-CoV-2 RNA between the control group and demeclocycline 150 and 300 mg groups (p = 0.343 and 0.333, respectively).

### T cell and cytokine responses after treatment

T cell and cytokine responses were analyzed from blood samples collected at baseline (prior to the study treatment on day 1) and on day 8. Blood samples collected on day 8 were obtained prior to the administration of dexamethasone, if applicable. Due to the administration of dexamethasone prior to blood sample collection on day 8, samples were not obtained from two patients in the control group, one in the demeclocycline 150 mg group, and two in the demeclocycline 300 mg group. Furthermore, a blood sample from one patient in the demeclocycline 300 mg group was not obtained due to patient withdrawal.

The average change rates of CD8^+^ T cells from day 1 to day 8 were 44.1% in the control group, 40.7% in the demeclocycline 150 mg group, and 57.4% in the demeclocycline 300 mg group (as shown in Table 3). The difference in the average change rate of CD8^+^ T cells between the control group and demeclocycline 150 mg group was -3.48% (95% CI − 81.95% to 74.99%), which was not significant (p = 0.539). Since no significant differences were observed between the control group and demeclocycline 150 mg group, a comparison of the average change rate of CD8^+^ T cells between the control group and demeclocycline 300 mg group was not performed according to the closed testing procedure. Regarding the average change in the CD8^+^ T cell count from day 1 to day 8, the differences between the control group and groups treated with demeclocycline at doses of 150 and 300 mg were 10.1/μL (95% CI − 149.6/μL to 169.8/μL) and 36.3/μL (95% CI − 225.0/μL to 297.6/μL), respectively (as shown in Table 3). These differences were not significant (p = 0.445 and p = 0.373, respectively). However, average changes in CD8^+^ T cells from day 1 to day 8 showed slight variations, particularly in the demeclocycline 150 mg group, which was recorded as 109.5/μL (95% CI − 1.6/μL to 220.6/μL; p = 0.026), whereas average changes were 135.7/μL in the demeclocycline 300 mg group (95% CI − 301.6/μL to 572.9/μL; p = 0.157) and 99.4/μL in the control group (95% CI − 60.5/μL to 259.3/μL; p = 0.080) (as shown in Table 3). Changes in HLA-DR^+^CD8^+^ T cell counts from day 1 to day 8 in the demeclocycline-treated groups were 71/μL, 54/μL, and 31/μL, respectively (Table 4). These results indicate that CD8^+^ T cells exhibited a weak response following the treatment with demeclocycline.

**Table 4:** Changes in HLA-DR^+^ T cells from day 1 to day 8 of the study treatment.

Arm		HLA-DR^+^CD8^+^			HLA-DR^+^CD4^+^		
		Day 1 (/µL)	Day 8 (/µL)	Change (/µL)	Day 1 (/µL)	Day 8 (/µL)	Change (/µL)
Demeclocycline		123	194	71	45	86	41
		110	164	54	49	102	53
		64	95	31	40	95	55
		218	–		57	–	
		58	–		50	–	
	Average			52.0 (N = 3)			49.7 (N = 3)

In contrast to CD8^+^ T cells, a greater number of disparities were observed in CD4^+^ T cells between the control group and demeclocycline-treated groups. The average change rates of CD4^+^ T cells from day 1 to day 8 were 57.0% in the demeclocycline 150 mg group and 68.5% in the demeclocycline 300 mg group, in contrast to only 32.4% in the control group (Table 3). Differences in the average change rate of CD4^+^ T cells between the control group and demeclocycline 150 and 300 mg groups were 24.55% (95% CI − 70.53% to 119.63%) and 36.05% (95% CI − 121.62% to 193.72%), respectively, which were not significant (p = 0.287 and p = 0.298, respectively). Regarding the average change in the CD4^+^ T cell count from day 1 to day 8, differences between the control group and the groups treated with demeclocycline at doses of 150 and 300 mg were 143.5/μL (95% CI − 89.4/μL to 376.4/μL) and 207.9/μL (95% CI − 189.9/μL to 605.6/μL), respectively (as shown in Table 3), which were not significant (p = 0.098 and p = 0.124, respectively). However, the average changes in CD4^+^ T cells from day 1 to day 8 significantly varied in the group treated with demeclocycline at a dose of 150 mg, which was recorded as 191.3/μL (95% CI 5.1/μL to 377.6/μL; p = 0.023), whereas average changes were 255.7/μL in the group treated with demeclocycline at a dose of 300 mg (95% CI − 519.0/μL to 1030.4/μL; p = 0.146) and 47.8/μL (95% CI − 151.2/μL to 246.8/μL; p = 0.271) in the control group (as shown in Table 3). Changes in HLA-DR^+^CD4^+^ T cell counts from day 1 to day 8 in the demeclocycline-treated groups were 55/μL, 53/μL, and 41/μL, respectively (Table 4). These results indicate that CD4^+^ T cells showed a response following the administration of demeclocycline. Furthermore, the change rates of CD4^+^ T cells in the demeclocycline-treated groups correlated with those of CD8^+^ T cells and CD19^+^ B cells (R = 0.831, p = 0.006, and R = 0.765, p = 0.016, respectively) (Supplementary Fig. 4).

**Figure 3:**
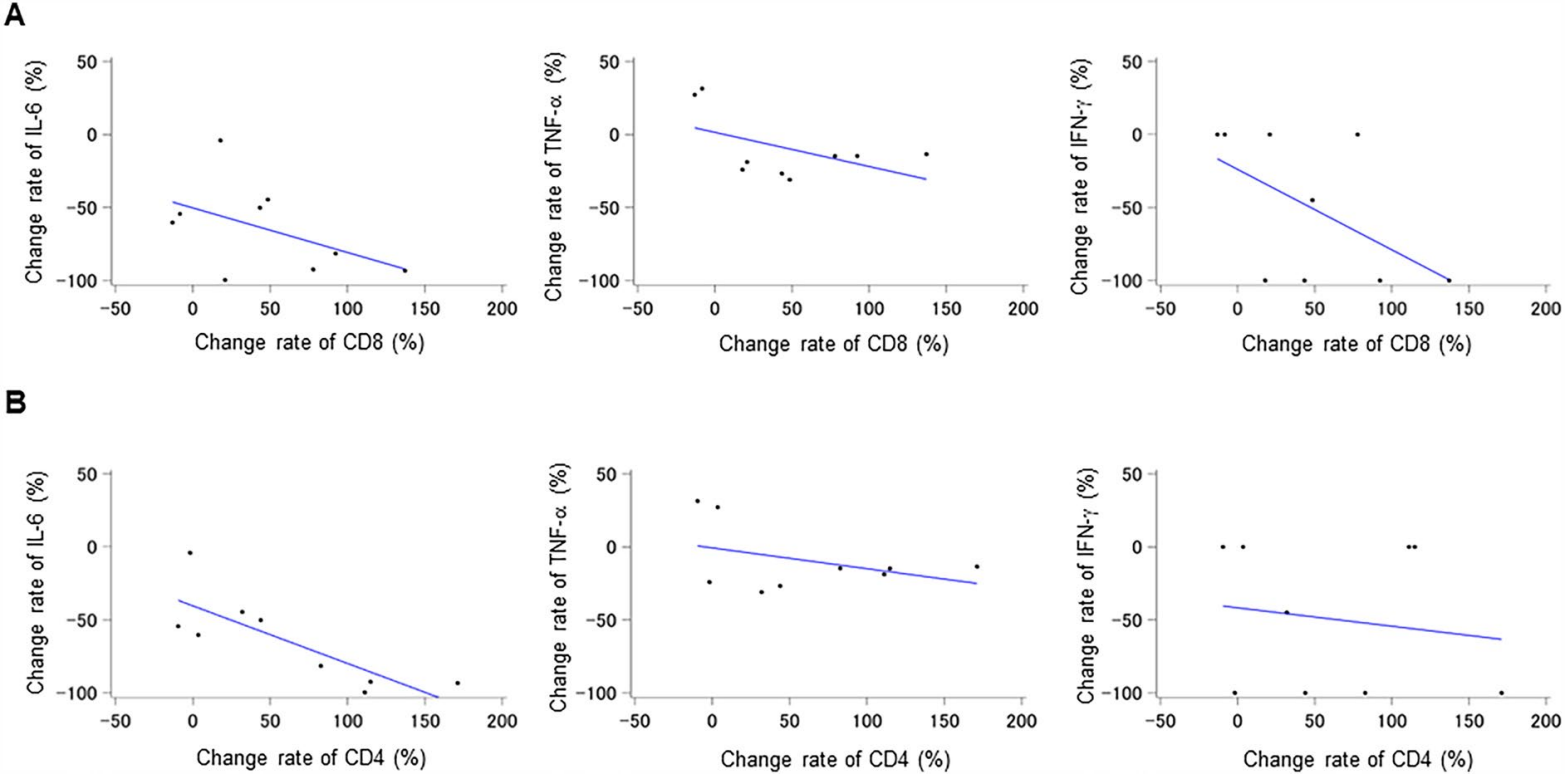
Relationships between change rates of T cells and cytokines in demeclocycline treatment groups (n = 9). (**A**) Correlations between the change rates of CD8^+^ T cells and cytokines (IL-6, TNF-α, and IFN-γ) in the demeclocycline treatment groups. (**B**) Correlations between the change rates of CD4^+^ T cells and cytokines (IL-6, TNF-α, and IFN-γ) in the demeclocycline treatment groups.

An analysis of the relationship between the change rates of T cells (CD4^+^ and CD8^+^) and cytokines (IL-6, TNF-α, and IFN-γ) in the demeclocycline-treated groups revealed no correlations, except for a negative correlation between the change rates of CD4^+^ T cells and IL-6 (R = -0.807, p = 0.009) (Fig. 3A and B). In conjunction with these results, patients treated with demeclocycline exhibited an increase in CD4^+^ T cell counts, which correlated with a decrease in IL-6 levels following treatment, suggesting the attenuation of COVID-19.

### B cell and antibody responses after treatment

The average change rates of CD19^+^ B cells from day 1 to day 8 were 45.58% in the demeclocycline 150 mg group, 91.86% in the demeclocycline 300 mg group, and 49.73% in the control group (Supplementary Fig. 5A). Differences in the average change rate of CD19^+^ B cells between the control group and demeclocycline 150 and 300 mg groups were − 4.15% (95% CI − 74.41% to 66.10%) and 42.12% (95% CI − 87.62% to 171.87%), respectively, which were not significant (p = 0.552 and p = 0.229, respectively). Differences in the average change in CD19^+^ B cells from day 1 to day 8 between the control group and the demeclocycline 150 and 300 mg groups were -7.9/μL (95% CI − 108.1/μL to 92.3/μL; p = 0.569) and 57.7/μL (95% CI − 103.0/μL to 218.5/μL; p = 0.207), respectively (Supplementary Fig. 5A). Differences in the number of CD19^+^ B cells from day 1 to day 8 were 64.6/μL (95% CI − 40.5/μL to 169.7/μL) in the control group, 56.7/μL (95% CI − 8.9/μL to 122.2/μL) in the demeclocycline 150 mg group, and 122.3/μL (95% CI − 125.4/μL to 370.0/μL) in the demeclocycline 300 mg group, which showed slight differences (p = 0.082, p = 0.038, and p = 0.084, respectively) (Supplementary Fig. 5B).

At the initiation of the study treatment, SARS-CoV-2 antibodies in three patients were positive. The durations from symptom onset to the initiation of the study treatment for these patients were seven, eleven, and thirteen days, respectively. Among patients whose antibodies were negative at treatment initiation, there was a 75.0% (3/4) positivity rate for SARS-CoV-2 antibodies in the control group, 83.3% (5/6) in the demeclocycline 150 mg group, and 100% (2/2) in the demeclocycline 300 mg group on day 8 of the study treatment. These results suggest that B cell counts and SARS-CoV-2 antibodies slightly increased in each group.

### COVID-19 symptoms

On day 1, the most prevalent symptom among each group was cough, with a prevalence of 57.1% (4/7) in the control group, 71.4% (5/7) in the demeclocycline 150 mg group, and 83.3% (5/6) in the demeclocycline 300 mg group (as shown in Supplementary Fig. 6). There was a reduction in the percentage of patients with cough from day 1 to day 15 across all groups. Additionally, there were patients reporting symptoms of chest pain, dyspnea on exertion, and dysgeusia on day 1 within each group. The percentage of patients with these symptoms also decreased from day 1 to day 15.

### Safety

Treatment-emergent adverse events (TEAEs) were reported by one (14.3%), one (14.3%), and three (50.0%) patients in the control, demeclocycline 150 mg, and demeclocycline 300 mg groups, respectively (as shown in Table 5). The severity of all TEAEs was mild to moderate. Notably, hyperuricemia was observed in four patients, all of whom were concurrently taking favipiravir. Treatment-related adverse events (TRAEs) were reported by zero (0.0%) and two (33.3%) patients in the demeclocycline 150 and 300 mg groups, respectively. The two TRAEs reported in the demeclocycline 300 mg group were rash and diarrhea.

**Table 5:** Adverse events (safety analysis population) over 29 days.

	Control group	Demeclocycline 150 mg group	Demeclocycline 300 mg group
Number of patients	7	7	6
Treatment-emergent adverse events (TEAEs), N (%)	1 (14.3%)	1 (14.3%)	3 (50%)
Hyperuricemia, event	1	1	2
Diarrhea, event	0	0	1
Rash, event	0	0	1
Treatment-related adverse events (TRAEs), N (%)	0 (0%)	0 (0%)	2 (33.3%)
Diarrhea, event	0	0	1
Rash, event	0	0	1
Severe adverse events	0 (0%)	0 (0%)	0 (0%)

## Discussion

In the present study, the administration of low-dose demeclocycline for patients with mild-to-moderate COVID-19 was well tolerated and demonstrated potential in mitigating the exacerbation of COVID-19. The number of CD4^+^ T cells significantly increased in individuals with mild-to-moderate COVID-19 who were administered 150 mg of demeclocycline on a daily basis. The change rate of CD4^+^ T cells in the demeclocycline treatment group positively correlated with those of CD8^+^ T cells and CD19^+^ B cells, and negatively correlated with IL-6 levels. These results suggest that patients with COVID-19 treated with demeclocycline exhibit immune responses indicative of recovery from the virus.

Patients with mild-to-moderate COVID-19 were recruited in the present study. Immunological responses varied based on the severity of the disease. In the RECOVERY trial, no clear benefit of the dexamethasone treatment was observed among patients with COVID-19 who did not require oxygen support. However, the mortality rate was lower among patients receiving oxygen support who were administered dexamethasone than in the group receiving standard care^14^. These findings may be partially attributed to the immunosuppressive effects of dexamethasone on immune responses in patients with mild-to-moderate COVID-19. Antiviral T cell responses play a vital role in the recovery of patients with mild-to-moderate COVID-19^16^. Therefore, based on previous findings, it was hypothesized that tetracyclines may be effective therapeutic agents for mild-to-moderate COVID-19, particularly regarding enhancements in T cell responses^24^.

In the present study, a significant increase was observed in the population of CD4^+^ T cells, including activated HLA-DR^+^CD4^+^ T cells, among patients with mild-to-moderate COVID-19 who were treated with demeclocycline. Previous studies indicated that CD4^+^ T cells, rather than CD8^+^ T cells, were the first to respond in patients with mild COVID-19 during the first two weeks of symptom onset^25^. Although CD4^+^ and CD8^+^ T cells both respond in patients with mild COVID-19^26,27^, it is possible that CD4^+^ T cells respond first, followed by CD8^+^ T cells. Furthermore, in the present study, the change rate in CD4^+^ T cells positively correlated with that in CD8^+^ T cells, but negatively correlated with that in IL-6, which is consistent with previous findings^28,29^. Additionally, IL-6 levels were shown to be elevated in patients with severe COVID-19 and decreased during the recovery phase of COVID-19^30^. These findings support the hypothesis that demeclocycline may be an effective treatment for COVID-19 by increasing the population of CD4^+^ T cells, which correlated with a decrease in IL-6.

A plethora of immunomodulatory agents, such as dexamethasone, balicitinib, tocilizumab, and sarilumab, are utilized to treat patients with severe COVID-19. However, there are currently no therapeutic agents that have been specifically developed to support antiviral T cell responses in patients with early and mild COVID-19. The findings of our previous preclinical studies demonstrated that tetracyclines enhanced antigen-specific T cell responses^24^. We are in the process of identifying the molecular target of tetracyclines in this mechanism of action, which has the potential to be applied to the treatment of not only COVID-19, but also other viral diseases.

The most frequent coexisting conditions in the present study were hypertension (20.0%) and diabetes mellitus (20.0%). Patients with hypertension were 2/7 (28.6%) in the control group and 2/7 (28.6%) in the demeclocycline 150 daily group. Patients with diabetes mellitus were also 2/7 (28.6%) in the control group and 2/7 (28.6%) in the demeclocycline 150 daily group. Hypertension and diabetes mellitus were reported to be at increased risk of developing severe COVID-19^31^. These coexisting conditions may affect the study results.

The present study had a number of limitations that need to be addressed. We did not conduct an analysis of SARS-CoV-2-specific T cells, which is crucial for understanding the immunological response to SARS-CoV-2 in patients with COVID-19. Furthermore, due to the small cohort size, it was challenging to evaluate clinical responses to the demeclocycline treatment. During the course of this clinical study, we encountered significant difficulties in both patient enrollment and study treatment completion. To minimize the effects of T cell responses, patients who had been vaccinated against COVID-19 were excluded from the study, and the concurrent use of corticosteroids, including dexamethasone, was prohibited during study treatment. Since the majority of Japanese citizens had rapidly received COVID-19 vaccines, there were a limited number of eligible COVID-19 patients who had not been vaccinated. Furthermore, all of the COVID-19 patients enrolled in this study were diagnosed with pneumonia via chest imaging and were generally indicated for dexamethasone treatments when demeclocycline treatments were terminated. We estimated that 8 patients were needed in each three groups, and actually, a total of 23 patients were randomized for the present study. However, the discontinuation of the study treatment, attributed to the administration of dexamethasone, resulted in a reduction in the cohort of patients assessed for T cell responses. Contrary to our hypothesis of CD8^+^ T cell responses, CD4^+^ T cells increased in the demeclocycline treated group. Nevertheless, a significant difference was not observed for the change rate of CD4^+^ T cells, as influenced by relatively small cohort size. Therefore, further large cohort studies are needed to confirm our results.

In summary, the present study suggests that patients with mild-to-moderate COVID-19 treated with low-dose demeclocycline exhibited T cell responses that were favorable for recovery from COVID-19. However, further studies with larger sample sizes are needed to confirm and expand upon these results.

## Methods

### Study design

The present study was an open-label, multicenter, parallel-group, randomized controlled clinical trial involving patients diagnosed with mild-to-moderate COVID-19. Participants with mild-to-moderate COVID-19 were planned to be randomly assigned in a 1:1:1 ratio to one of three groups: a control group (normal treatment), a group receiving 150 mg of demeclocycline once daily, and a group receiving 300 mg of demeclocycline (150 mg twice daily). Individuals allocated to the demeclocycline groups received a daily dose of 150 mg once daily or 150 mg twice daily for a period of 14 days.

This clinical trial was conducted in accordance with the guidelines set forth in the Declaration of Helsinki. The study protocol received approval from the Certified Review Board of Osaka University (CRB5180007) and each individual participating institution. Written informed consent was obtained from patients prior to their inclusion in the study. This study was registered with the Japan Registry of Clinical Trials as jRCTs051200049 (https://jrct.niph.go.jp/en-latest-detail/jRCTs051200049).

### Patients

We prospectively identified patients aged between 20 and 75 years with SARS-CoV-2 infection confirmed by a positive reverse transcription-polymerase chain reaction (RT-PCR) or SARS-CoV-2 antigen tests that were approved by the Ministry of Health, Labour and Welfare in Japan. Inclusion criteria were mild-to-moderate COVID-19 without the need for oxygen support. Exclusion criteria were an SpO_2_ concentration < 93% at room air and any COVID-19 vaccination prior to randomization.

### Treatment

Patients in the demeclocycline 150 mg daily group and demeclocycline 300 mg daily group (administered as 150 mg twice daily) received the study drug orally in the form of demeclocycline 150 mg capsules for a period of 14 days. The concurrent use of corticosteroids, including dexamethasone, immunosuppressants, and immunostimulants, or biologics, such as vaccines and antibody drugs, was prohibited during the study treatment, except for sotrovimab or casirivimab plus imdevimab. If a patient required the administration of dexamethasone due to the exacerbation of COVID-19, the study treatment was discontinued.

### Randomization

The patients were randomly assigned to one of three groups; control, demeclocycline 150 mg daily, demeclocycline 300 mg daily groups. The allocation ratio was set at 1:1:1. We used stratified randomization by institute with permuted blocks (block size = 3). In this study, an open-label design was applied. The reason for this design was that the primary endpoint was the enhancement of the T cell response, which is an objective measure devoid of evaluator bias. The statistician, who was responsible for this study, generated the allocation list confidentially and implementation was managed via the electronic data capture system (Datatrak Enterprise Cloud^®^).

### Outcomes and assessments

The primary and secondary outcomes of the present study included changes from baseline (day 1, prior to the study treatment) in T cells, cytokines, and SARS-CoV-2 RNA on day 8. The primary outcome was the change rate of peripheral CD8^+^ T cells. Secondary outcomes included the change in CD8^+^ T cells, change rates of CD4^+^ T cells, CD19^+^ B cells, cytokines (IL-6, TNF-α, and IFN-γ), and the rate of the negative conversion of SARS-CoV-2 RNA. The measurement of CD4 (anti-CD4-FITC, clone SK3, Becton Dickinson), CD8 (anti-CD8-FITC, clone SK1, Becton Dickinson), and CD19 (anti-CD19-FITC, clone B4, Beckman Coulter) was performed using FACS Calibur (Becton Dickinson) at LSI Medience (Tokyo, Japan). In the middle of the study, to analyze activated T cells, the measurement of HLA-DR (anti-HLA-DR-PE, clone L243, Becton Dickinson) and CD4 (anti-CD4-FITC, clone SK3, Becton Dickinson) or CD8 (anti-CD8-FITC, clone SK1, Becton Dickinson) was also conducted using FACS Calibur (Becton Dickinson) at LSI Medience (Tokyo, Japan). These lymphocytes were measured at room temperature within 24 h of blood sample collection. Plasma IL-6 concentrations were measured using the Quanti Glo ELISA Human IL-6 Immunoassay kit (R&D Systems), plasma TNF-α concentrations with the QuantiGlo ELISA Human TNF-α Immunoassay kit (R&D Systems), and plasma IFN-γ concentrations with the Human IFN Gamma Platinum ELISA kit (Thermo Fisher Scientific). RT-PCR for SARS-CoV-2 in saliva samples was performed using the LightMix Modular SARS-CoV (COVID-19) E-gene kit (Roche Diagnostics). Serum SARS-CoV-2 antibodies were measured using the Elecsys Anti-SARS-CoV-2 kit (Roche Diagnostics). The measurement of cytokines (IL-6, TNF-α, and IFN-γ), SARS-CoV-2 RT-PCR, and SARS-CoV-2 antibodies was performed at LSI Medience (Tokyo, Japan).

Clinical outcomes included the change in oxygen saturation measured by pulse oximetry (SpO_2_), NEWS, the seven-category ordinal scale, and nine COVID-19 symptoms.

### Sample size and statistical analyses

We estimated that 8 patients were needed in each group to detect a 15% difference in the change rate of CD8^+^ cells between the control and demeclocycline 150 mg groups with Student’s t-test, a two-sided 5% significance level and 80% power. To determine the effect size (a 15% difference), we drew upon non-clinical experimental data and referenced the article by Wang et al.^18^. The rationale behind conducting sample size calculations solely for the comparative analysis between the 150 mg group and the control group, despite the allocation to three groups, stems from the implementation of a fixed sequence procedure to account for multiplicity adjustment.

Efficacy analyses were performed on the mITT population, where patients without receiving the study treatment at any time, without any observations on efficacy/safety, or with eligibility violations were excluded from all randomized patients.

The primary outcome, a difference in the change rate of CD8^+^ cells from day 1 (baseline, prior to the study treatment) to day 8 (seven days after the study treatment) between groups (demeclocycline treated group—control group) was tested using the Student’s *t*-test. Following the fixed sequence procedure, we compared the control and demeclocycline 150 mg groups, and if the result was significant, further comparisons were performed with the demeclocycline 300 mg group.

Regarding secondary outcomes, differences in the change in or change rate of T cells (CD8^+^, CD4^+^) and CD19^+^ B cells among groups were tested using the Student’s *t*-test. We also performed a paired *t*-test in each group to confirm the change in cells from day 1 to day 8. We examined changes in HLA-DR^+^CD8^+^ and HLA-DR^+^CD4^+^ from day 1 to day 8 in some patients. Regarding changes in SpO_2_ and NEWS, the Student’s *t*-test was used to examine the significance of differences. In survival data (the time before oxygen therapy or dexamethasone administration), the cumulative incidence was estimated using the Kaplan–Meier method and the Log-rank test was performed. In count data (SARS-CoV-2 viral RNA level and the SARS-CoV-2 antibody), we calculated the percentage of negative/positive patients on day 8 in the numerator and positive/negative patients on day 1 in the denominator, respectively. We also performed Fisher’s exact test to compare percentages among groups. Regarding clinical outcomes (the seven-category ordinal scale), number counts and percentage calculations were performed. To investigate the relationships between T cells and cytokines (IL-6, TNF-α, and IFN-γ), Pearson’s product moment correlation coefficient was calculated between T cells and cytokines (IL-6, TNF-α, and IFN-γ). All tests were set at the one-sided significance level of 0.025, except for correlation tests with a two-sided significance level of 0.05.

The safety assessment was based on adverse events reported during this trial. We calculated the number and percentage of all adverse events, which were coded according to Medical Dictionary for Regulatory Activities (MedDRA) terms.

All analyses were conducted with SAS Version 9.4 software (SAS Institute, Inc., Cary, NC).

### Supplementary Information

Supplementary Information.

## Data Availability

The authors declare that the primary data supporting the results of the present study are available within the article and its supplementary information files. Additional data may be obtained from the corresponding author upon reasonable request.
