# Supplementary material for: A randomized phase 2 study on demeclocycline in patients with mild-to-moderate COVID-19
Source: Sci Rep. 2023 Aug 23;13:13809. doi: 10.1038/s41598-023-41051-2 (PMC10447520; doi:10.1038/s41598-023-41051-2)
Supplement: Supplementary file 1 — Supplementary Information. [file 41598_2023_41051_MOESM1_ESM.pdf]

A randomized phase 2 study on demeclocycline in patients with mild-to-moderate COVID-19.

Kota Iwahori,[1,2]* Takuro Nii,[3] Norihiko Yamaguchi,[4] Takahiro Kawasaki,[1] Satomi Okamura,[5] Kazuki Hashimoto,[3] Takanori Matsuki,[3] Kazuyuki Tsujino,[3] Keisuke Miki,[3] Akio Osa,[4] Sho Goya,[4] Kinya Abe,[6] Masahide Mori,[7] Yoshito Takeda,[1] Tomomi Yamada,[5] Hiroshi Kida,[3] Atsushi Kumanogoh[1,8,9.10.11,12]

1 Department of Respiratory Medicine and Clinical Immunology, Graduate School of Medicine, Osaka University, Osaka, Japan.

2 Department of Clinical Research in Tumor Immunology, Graduate School of Medicine, Osaka University, Osaka, Japan.

3 Department of Respiratory Medicine, National Hospital Organization Osaka Toneyama Medical Center, Osaka, Japan.

4 Department of Respiratory Medicine, Kinki Central Hospital of Mutual Aid Association of Public School Teachers, Hyogo, Japan.

5 Department of Medical Innovation, Osaka University Hospital, Osaka, Japan.

6 Department of Internal Medicine, Toyonaka Municipal Hospital, Japan.

7 Department of Thoracic Oncology, National Hospital Organization Osaka Toneyama Medical Center, Osaka, Japan.

8 Department of Immunopathology, World Premier International Research Center Initiative (WPI), Immunology Frontier Research Center (IFReC), Osaka University, Suita, Japan.

9 Integrated Frontier Research for Medical Science Division, Institute for Open and Transdisciplinary Research Initiatives (OTRI), Osaka University, Osaka, Japan.

10 Center for Infectious Diseases for Education and Research (CiDER), Osaka University, Suita, Japan.

11 Japan Agency for Medical Research and Development – Core Research for Evolutional Science and Technology (AMED–CREST), Osaka University, Osaka, Japan

12 Center for Advanced Modalities and DDS (CAMaD), Osaka University, Osaka, Japan

KI, TN, and NY contributed equally to this work.

*To whom correspondence should be addressed:

Kota Iwahori

Department of Respiratory Medicine and Clinical Immunology, Graduate School of Medicine, Osaka University, 2-2 Yamadaoka, Suita, Osaka 565-0871, Japan.

E-mail address: iwahori@climm.med.osaka-u.ac.jp

**Supplementary Table 1.** Concurrent treatments in each group.

| | | Total | Control group | Demeclocycline 150 mg group | Demeclocycline 300 mg group |
|---|---|---|---|---|---|
| Number of patients | | 20 | 7 | 7 | 6 |
| Concurrent treatment | Casirivimab-Imdevimab | 6 | 2 | 2 | 2 |
| | Remdesivir | 3 | 1 | 0 | 2 |
| | Favipiravir | 14 | 4 | 5 | 5 |
| | Ciclesonide | 2 | 1 | 1 | 0 |

**Supplementary Table 2.** The NEWS score between day 1 and day 29.

| | | Day 1 | Day 8 | Day 15 | Day 29 |
|---|---|---|---|---|---|
| Control group | Number of patients | 6 | 4 | 1 | 1 |
| | Mean | 0.8 | 1.0 | 0.0 | 1.0 |
| | SD | 1.0 | 0.8 | - | - |
| Demeclocycline 150 mg group | Number of patients | 6 | 4 | 2 | 1 |
| | Mean | 1.2 | 1.3 | 1.5 | 0.0 |
| | SD | 1.2 | 1.3 | 0.7 | - |
| Demeclocycline 300 mg group | Number of patients | 5 | 4 | 2 | 1 |
| | Mean | 2.4 | 1.0 | 1.5 | 1.0 |
| | SD | 1.8 | 0.8 | 0.7 | - |

**Supplementary Table 3.** The NEWS scoring system.

| | Score | | | | | | |
|---|---|---|---|---|---|---|---|
| | 3 | 2 | 1 | 0 | 1 | 2 | 3 |
| Respiration rate (per minute) | ≤ 8 | | 9 - 11 | 12 - 20 | | 21 - 24 | ≥ 25 |
| $SpO_2$ scale (%) | ≤ 91 | 92 -93 | 94 - 95 | ≥ 96 | | | |
| Air or oxygen | | Oxygen | | Air | | | |
| Systolic blood pressure (mmHg) | ≤ 90 | 91 - 100 | 101 - 110 | 111 - 219 | | | ≥ 220 |
| Pulse (per minute) | ≤ 40 | | 41 - 50 | 51 - 90 | 91 - 110 | 111 - 130 | ≥ 131 |
| Consciousness | | | | Alert | | | CVPU |
| Temperature (°C) | ≤ 35.0 | | 35.1 - 36.0 | 36.1 - 38.0 | 38.1 - 39.0 | ≥ 39.1 | |

CVPU: confusion, verbal, pain, unresponsive.

**Supplementary Figure 1**

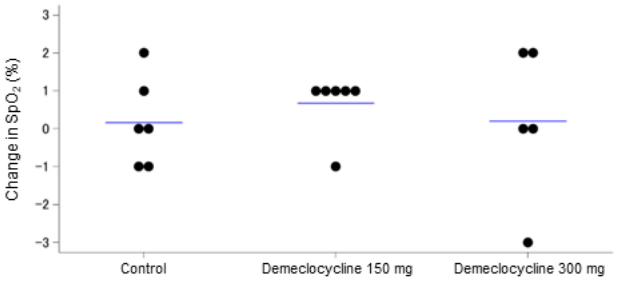


**Supplementary Figure 1.** Changes in $SpO_2$ from day 1 to day 8 of the study treatment in the control group (n = 6), demeclocycline 150 mg group (n = 6), and demeclocycline 300 mg group (n = 5).

## Supplementary Figure 2

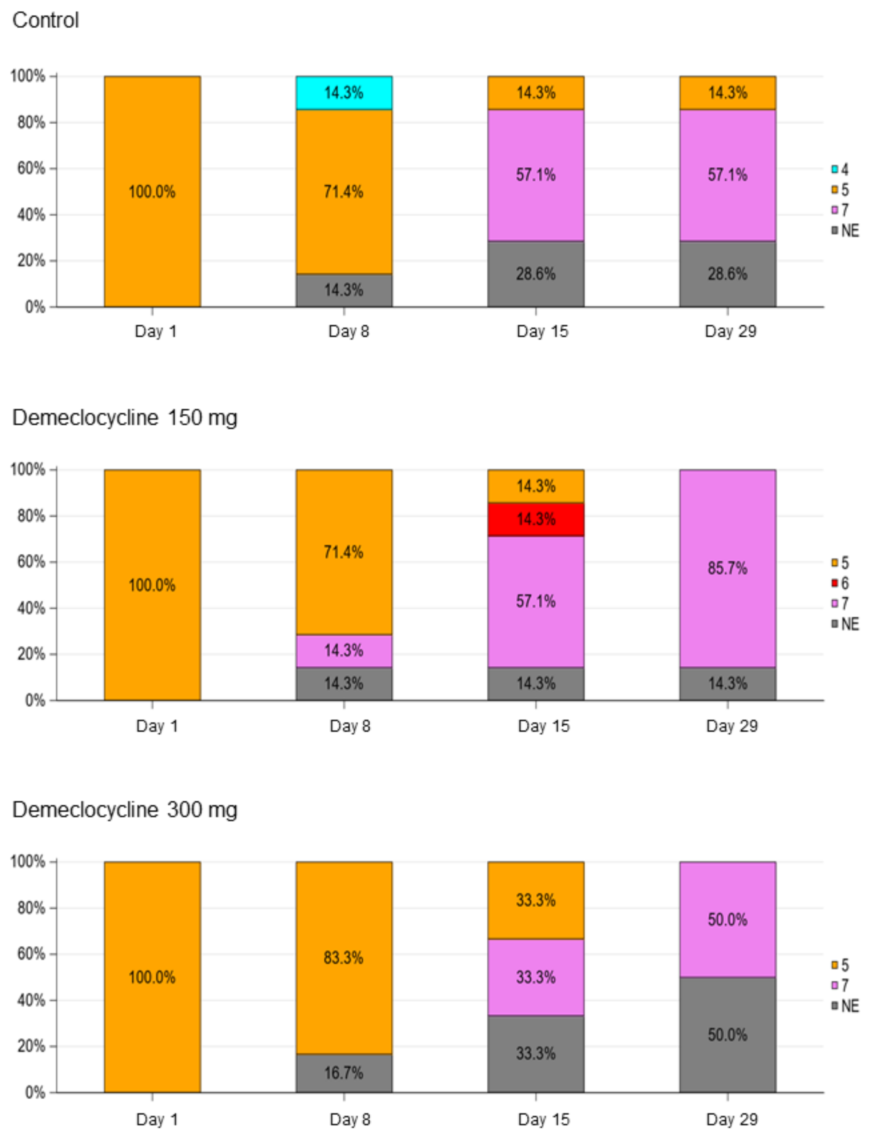


Seven-category ordinal scale

1 death
2 hospitalized, requiring ECMO, invasive mechanical ventilation, or both
3 hospitalized, requiring nasal high-flow oxygen therapy or non-invasive mechanical ventilation
4 hospitalized, requiring supplemental oxygen
5 hospitalized, not requiring supplemental oxygen
6 hospitalized, not requiring treatment without the study treatment
7 discharged without any treatment

**Supplementary Figure 2.** Clinical status, defined by a seven-category ordinal scale, between day 1 and day 29 in the control group (n = 7), demeclocycline 150 mg group (n = 7), and demeclocycline 300 mg group (n = 6). NE, not evaluable.

**Supplementary Figure 3**

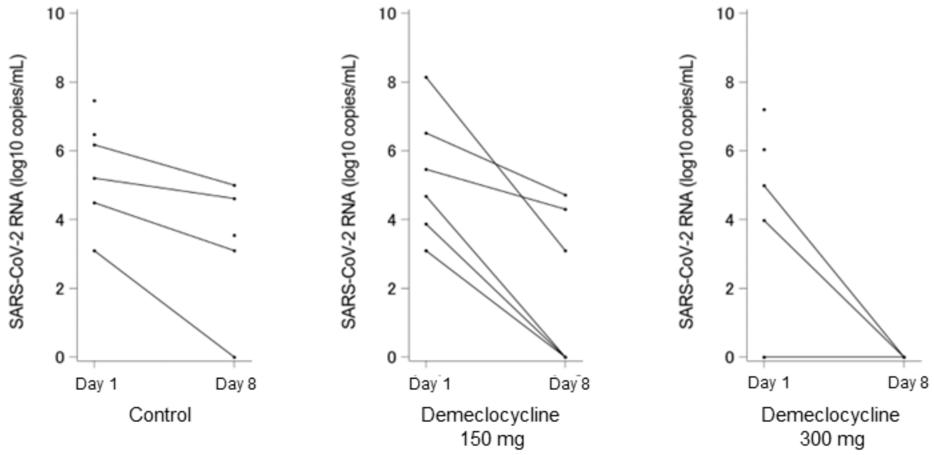


**Supplementary Figure 3.** Changes in SARS-CoV-2 RNA from day 1 to day 8 of the study treatment in the control group (n = 4), demeclocycline 150 mg group (n = 6), and demeclocycline 300 mg group (n = 3).

**Supplementary Figure 4**

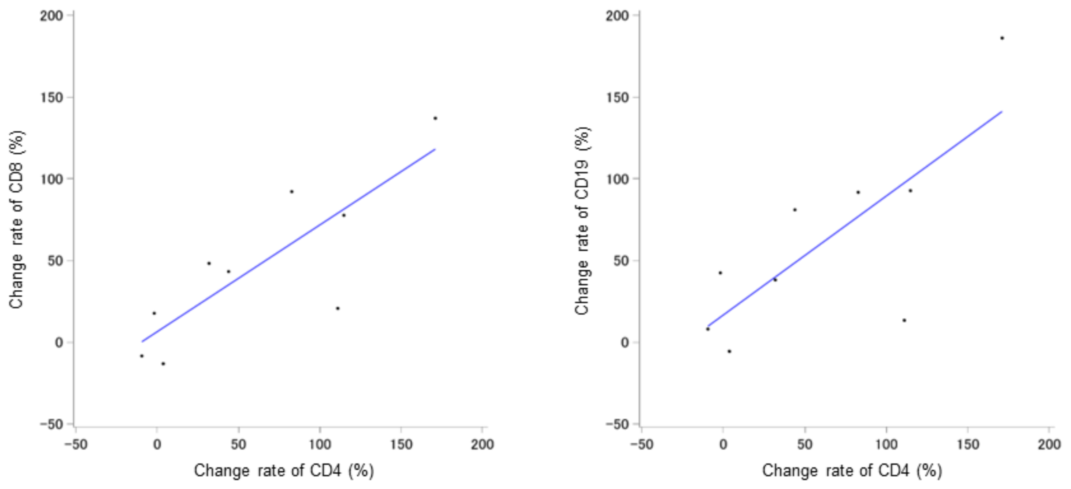


**Supplementary Figure 4.** Relationships between change rates of CD4+ and CD8+ T cells and between CD4+ T cells and CD19+ B cells in demeclocycline treatment groups (n = 9).

**Supplementary Figure 5**

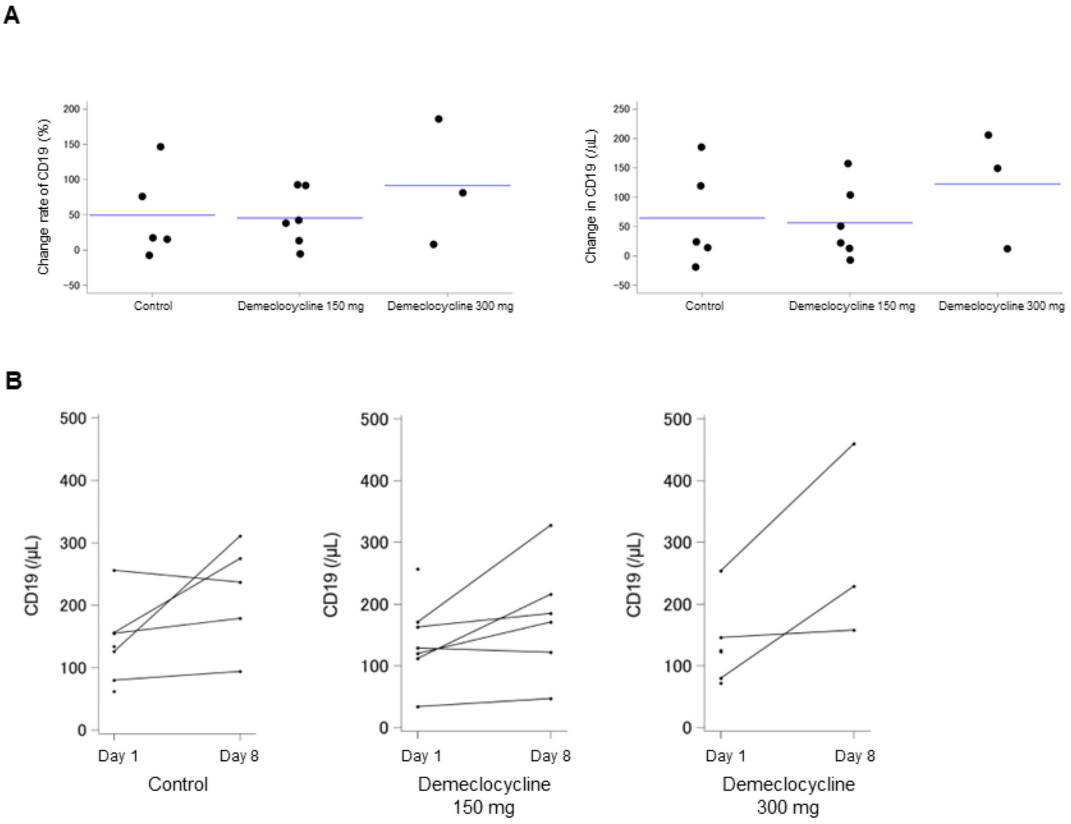


**Supplementary Figure 5.** (A) Changes in (right) and change rates (left) of CD19+ B cells in the control group (n = 5), demeclocycline 150 mg group (n = 6), and demeclocycline 300 mg group (n = 3). Each bar represents the average for each group. (B) Changes in CD19+ B cells from day 1 to day 8 of the study treatment in the control group (n = 5), demeclocycline 150 mg group (n = 6), and demeclocycline 300 mg group (n = 3).

**Supplementary Figure 6**

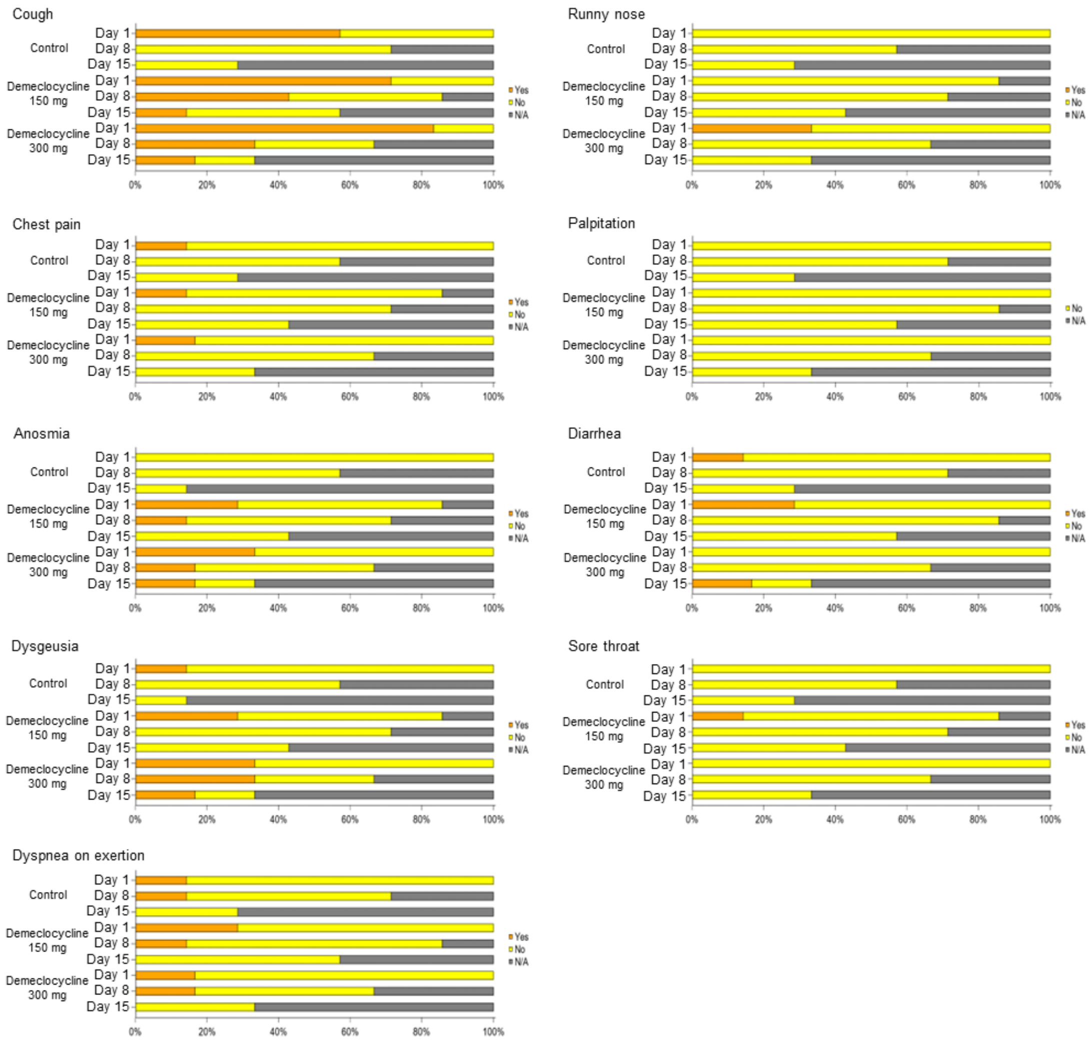


**Supplementary Figure 6.** The incidence of nine COVID-19 symptoms between day 1 and day 15 in the control group (n = 7), demeclocycline 150 mg group (n = 7), and demeclocycline 300 mg group (n = 6). N/A, not available.
